# Cues to Opening Mechanisms From *in Silico* Electric Field Excitation of Cx26 Hemichannel and *in Vitro* Mutagenesis Studies in HeLa Transfectans

**DOI:** 10.3389/fnmol.2018.00170

**Published:** 2018-05-31

**Authors:** Francesco Zonta, Damiano Buratto, Giulia Crispino, Andrea Carrer, Francesca Bruno, Guang Yang, Fabio Mammano, Sergio Pantano

**Affiliations:** ^1^Shanghai Institute for Advanced Immunochemical Studies, ShanghaiTech University, Shanghai, China; ^2^CNR Institute of Cell Biology and Neurobiology, Monterotondo, Italy; ^3^Department of Physics and Astronomy “G. Galilei”, University of Padua, Padua, Italy; ^4^Venetian Institute of Molecular Medicine, Padua, Italy; ^5^Department of Biomedical Sciences, University of Padua, Padua, Italy; ^6^Group of Biomolecular Simulations, Institut Pasteur de Montevideo, Montevideo, Uruguay

**Keywords:** gap junction hemichannels, coarse grained simulations, connexin, electrophysiology, Cx26, SIRAH force field, conformational change

## Abstract

Connexin channels play numerous essential roles in virtually every organ by mediating solute exchange between adjacent cells, or between cytoplasm and extracellular milieu. Our understanding of the structure-function relationship of connexin channels relies on X-ray crystallographic data for human connexin 26 (hCx26) intercellular gap junction channels. Comparison of experimental data and molecular dynamics simulations suggests that the published structures represent neither fully-open nor closed configurations. To facilitate the search for alternative stable configurations, we developed a coarse grained (CG) molecular model of the hCx26 hemichannel and studied its responses to external electric fields. When challenged by a field of 0.06 V/nm, the hemichannel relaxed toward a novel configuration characterized by a widened pore and an increased bending of the second transmembrane helix (TM2) at the level of the conserved Pro87. A point mutation that inhibited such transition in our simulations impeded hemichannel opening in electrophysiology and dye uptake experiments conducted on HeLa tranfectants. These results suggest that the hCx26 hemichannel uses a global degree of freedom to transit between different configuration states, which may be shared among the whole connexin family.

## Introduction

Connexins are topologically conserved proteins composed of four transmembrane (TM) helixes connected by two extracellular loops (EC1, EC2) and one cytoplasmic (CL) loop. Connexin hemichannels, also known as connexons, are hexameric arrays of connexins. They can function as regular membrane channels (Bennett et al., 2003; Sáez and Leybaert, 2014), or dock in the extracellular space to form gap junction channels that support direct cell-to-cell communication by connecting adjacent cytoplasmic spaces (Goodenough and Paul, 2009). Gap junction channels cluster in plaques by the thousands, and have been identified in virtually all tissues (Evans and Martin, 2002). It has been estimated that over 35.000 members of the human metabolome can be transferred through gap junction channels, or exchanged through unpaired connexons (Esseltine and Laird, 2016). In recent years, it has been discovered that also small, non-coding RNA (siRNAs or miRNAs) can diffuse through connexin-made channels (Esseltine and Laird, 2016). The relevance of connexins and their tightly regulated function is highlighted by their implication in pathological states of completely different character, such as cancer (Tsai et al., 2018), inflammation (Li et al., 2018), and neurodegenerative diseases (Belousov et al., 2018). For this reason, modulation of connexin hemichannels is becoming increasingly interesting for the treatment of several diseases including Alzheimer Disease (Yi et al., 2017), skin disorders, or X-linked Charcot Marie Tooth disease (Sáez and Leybaert, 2014; Carrer et al., 2017; Xu et al., 2017).

The only X-ray structures available at atomistic resolution for the connexin protein family are those corresponding to Ca^2+^-free (Maeda et al., 2009; Bennett et al., 2016) and Ca^2+^-bound (Bennett et al., 2016) forms of the hCx26 gap junction channel. The two structures, which have been associated with conductive and non-conductive states, respectively, overlap almost completely. For this reason, it has been recently hypothesized that the non-conductive state is associated with an electrostatic barrier created by bound calcium ions in the outer vestibule of the connexon (Bennett et al., 2016). However, this explanation meets some difficulties: (i) in the Ca^2+^-free configuration the pore seems not wide enough to allow the passage of small non-coding RNAs or relatively large molecules such as ATP, NADP, prostaglandins, etc., which are rapidly released from the cytoplasm to mediate autocrine/paracrine signaling (Kar et al., 2012); (ii) in the Ca^2+^-bound configuration, the pore is too wide to prevent the flux of small neutral molecules, such as water or glucose, that interact negligibly with the electrostatic barrier and therefore would diffuse freely through undocked connexons. Moreover: (iii) atomic force microscopy identified a significantly enlarged conformation of the extracellular mouth of the connexon in the Ca^2+^-free state (Müller et al., 2002); (iv) spontaneous transitions between non-conductive and conductive states of Cx32 and Cx26 hemichannels have been observed by electrophysiology and dye-uptake experiments under resting conditions (Fasciani et al., 2013). Indeed, single Cx26 hemichannel recordings show frequent alternation between conductive and non-conductive states at TM potentials (Δ*V*_*m*_, inside minus outside) ≤ −30 mV even in zero extracellular Ca^2+^ concentration ([Ca^2+^]_o_) (Sanchez et al., 2013, 2016). This behavior has been attributed to the so-called “loop gate” of Cx26, which is thought to remain constitutively active at all voltages (Sanchez et al., 2016). The same behavior is not observed in hemichannels formed by other connexins (e.g., connexin 46 and 50), which remain stably open in low [Ca^2+^]_o_ and close only if subjected to robust hyperpolarization (Trexler et al., 1996; Srinivas et al., 2005). Finally, (v) early molecular dynamics simulations questioned whether the published structures represent a fully open channel, since permeation and conductance properties could be reproduced only partially(Kwon et al., 2011; Zonta et al., 2012).

Our working hypothesis, based on the above considerations, is that connexin hemichannels may undergo yet uncharacterized conformational transitions, which we sought to explore using theoretical methods. Several approaches have been implemented to the study of membrane proteins mimicking a cell membrane potential (Cohen and Venkatachalam, 2014) using MD simulations (Delemotte et al., 2011; Bernardi et al., 2015; Escalona et al., 2016; Villanelo et al., 2017). A comprehensive review of the different theoretical approaches that have contributed to the study of connexin channels has been recently published (Villanelo et al., 2017). In particular, it has been recently shown that relatively high electric field pulses in combination with time-resolved X-ray crystallography can unravel details on the conformation and mechanics of proteins (Hekstra et al., 2016). Inspired by this technique, we used coarse-grained (CG) molecular dynamics using the SIRAH force field (Darré et al., 2015) to explore the conformational behavior of the hCx26 connexon in the presence and absence of an external field aligned with the pore axis. This computational technique applies a force perpendicular to the membrane to all charged particles. Because of the features of the SIRAH force field, all but hydrophobic CG particles beads carry a (partial) charge. Hence, the electric field will impact on all degrees of freedom with non-zero components along the membrane perpendicular. In this sense, we can expect the electric field to act like a biasing potential, in rough analogy to enhanced sampling methods used in combination with molecular dynamics (Grübmuller, 1995; Laio and Parrinello, 2002). Applying an electric field to a CG membrane protein allowed us to speed up simulations significantly while avoiding the use of *ad-hoc* biases and keeping a more realistic explicit representation of the membrane and solvent environment.

## Materials and methods

CG simulations were performed using the SIRAH force field (Darré et al., 2015) (www.sirahff.com), which is sensitive to variations in ionic strength and protein sequence (Surdo et al., 2017), and the lipid parameterization presented in Astrada et al. (2016).

The starting positions for the atoms of the Ca^2+^ free hCx26connexon were obtained from our previously published all-atom (AA) model (Zonta et al., 2012, 2013, 2014, 2015), and comprised also the amino acids not present in the experimental structure (such as the intracellular loop). Following the same procedure, we generated a model of the Ca^2+^-bound conformation using the X-ray structure by Bennett et al. (2016) as template for the TM region, and that of Maeda et al. (2009) for the part that were not resolved in the former. These AA models were converted to CG using SIRAH tools (Machado and Pantano, 2016) and inserted in a square membrane patch containing 230 phospholipid (DMPC) molecules. The systems were then solvated with 2377 CG solvent molecules called WT4 (Darre et al., 2010), comprising 115 Chloride, 31 Potassium, and 30 Sodium ions, altogether accounting for an ionic strength of approximately 150 mM. For charge neutralization, six Potassium and six Sodium ions were removed from the Ca^2+^-bound system. Ca^2+^ parameters were the same recently introduced and tested in Cali et al. (2017). After stabilization, the simulation box converged to sizes of 9.9, 9.9, and 13.5 nm for x, y, and z coordinates, respectively.

The simulation protocol for CG simulations consisted of 5,000 steps of unconstrained energy minimization followed by 10 ns of MD performed in the presence of positional constraints of 1,000 kJ/mol/nm^2^ on all the protein beads with a time step of 10 fs. This was followed by 10 ns of unconstrained simulation with a time step of 15 fs. Production runs were performed for at least 1 μs. All CG simulations were performed in the NPT ensemble at 300 K coupling protein, phospholipids and water/ions to three separate v-rescale thermostats (Bussi et al., 2007). Pressure was fixed at 1 bar using semi anisotropic coupling using Parrinello-Rahman barostats (Parrinello and Rahman, 1981). A timestep of 20 fs and a direct cutoff for non-bonded interactions of 1.2 nm was used. Long-range electrostatics was calculated with Particle Mesh Ewald summation method (Darden et al., 1993). All simulations were performed with Gromacs 4.6.7 (Pronk et al., 2013).

When indicated, an external electric field was applied in the direction perpendicular to the membrane plane (arbitrarily chosen as the Z direction) as implemented in Gromacs. Backmapping of CG conformers of wild type (wt) hCx26 and its T86L mutant was performed using SIRAH tools (Machado and Pantano, 2016).

### Calculated properties

Root mean square deviations were calculated on Cα atoms (or beads) in all cases. According to our previous results, the maximum constriction point of the channel is located at the level of Lys41 (Zonta et al., 2012). Hence, the minimum diameter of the pore was estimated as the average distance on the three pairs of opposed Lys 41 minus the van der Waals (vdW) radii of each bead (0.55 nm; Darré et al., 2015). The kink angle at TM2 was measured as the angle formed between the Cα atoms of amino acids Gln80, Pro87, and His94, i.e., two helix turns before and after the kink introduced by Pro87.

### Cx26T86L mutagenesis

The QuikChange II Site-Directed Mutagenesis Kit (Agilent, cat. no. 200523) was used to mutagenize the Threonine in position 86 of wt hCx26 to Leucine using the Cx26WTVenus pcDNA3.1 construct (Beltramello et al., 2005) as template. The primers were the followings:

Cx26T86Lf: 5′–CTGATCTTCGTGTCCCTGCCAGCGCTCCTAGTG−3′

Cx26T86Lr: 5′–CACTAGGAGCGCTGGCAGGGACACGAAGATCAG−3′. The correct insertion of the mutation was verified by DNA Sanger Sequencing.

### Patch clamp and dye-uptake assays

HeLa DH cells (Sigma-Aldrich, Cat. No. 96112022) were seeded onto round glass coverslips (Fisher Scientific, Cat. No. FIS#12-542A). Cells were maintained in Dulbecco's modified Eagle's medium (ThermoFisher, Cat.No. 41965039) containing 10% (v/v) FBS (Gibco-Invitrogen, Cat.No. 10270-106) and 1% penicillin/streptomycin (Gibco-Invitrogen, Cat.No. 15070-063). Twenty four hours after plating, a transfection reagent (Lipofectamine 2000, Cat. No. 11668-019) was used to transiently transfect cells at 25–30% confluence with wild type hCx26–Venus construct or its T86L mutant.

A double stage vertical puller (PP-830, Narishige) was used to fabricate patch pipettes from glass capillaries (G85150T-4, Harvard Apparatus, Edenbridge, UK). Pipettes were filled with a potassium aspartate (KAsp)-based intracellular solution (ICS_KAsp_) containing (in mM): 115 KAsp, 10 NaCl, 10 KCl, 1 MgCl_2_, 10 HEPES, 1 CaCl_2_, and 5 BAPTA tetrapotassium salt (pH 7.2, 311 mOsm) and filtered through 0.22-mm pores (Millipore). Filled pipettes had resistances of 4-6 MΩ when immersed in a NaCl-based extracellular solution (ECS_NaCl_) containing a reduced (0.2 mM) Ca^2+^ concentration ([Ca^2+^]_0_) and (in mM): 140 NaCl, 5 KCl, 10 HEPES, 2 sodium pyruvate, 4 tetraethylammonium chloride (TEA-Cl), 4 CsCl and 5 glucose (pH 7.4, 323 mOsm).

Twenty four hours after transfection, glass coverslips with adherent cells were transferred to the stage of an upright fluorescence microscope (BX51, Olympus) equipped with differential interference contrast (DIC) optics. Cells were continuously superfused at 2 ml/min at 20–23°C with ECS_NaCl_. Hemichannel currents were assayed in ECS_NaCl_ while keeping cells near their zero-current potential (between −20 and 0 mV) under whole cell patch clamp recording conditions. Cells were transiently depolarized to +40 mV for 20 s followed by a 1 s ramp down −40 or −60 mV and subsequently held at this negative potential for up to 1 min before stepping back to the zero-current potential. Alternatively, they were stimulated with slow (8 min) voltage ramps from +60 to −60 mV. To measure junctional currents, pairs of HeLa DH cells visibly connected by a gap junction plaque were selected, patch clamped with two separate amplifiers and initially held at 0 mV. Then one of the two cells (cell 1) was stimulated by voltage commands while recording the whole cell current from the unstimulated cell (cell 2). For dye uptake assays, HeLaDH cells were seeded at 40% confluence and transfected 24 h later with Cx26Venus constructs (wt and T86L). Control cells were treated with Lipofectamine 2000 alone.

Six hours after transfection, cells were washed three times with D-PBS (Thermo Fisher Scientific, cat. no.14190250), incubated at room temperature for 1 h with propidium iodide (PI, ThermoFisher, Cat No. P1304MP, 0.25 mM dissolved in D-PBS). Cells were then washed in HBSS, fixed in PFA 2%, mounted onto glass slides with a mounting medium (FluorSave™ Reagent, cat. 345789, Merk Millipore) and observed using a confocal microscope (TCS SP5, Leica) equipped with an oil–immersion objective (63 × HCX PL APO 1.4 N.A., Leica). Samples were excited with Argon laser 488 (Venus) and 561 (PI) lines.

## Results

### CG molecular dynamics of the hCx26 connexon reproduce atomistic simulations in the absence of external electric fields

**Figure 1:**
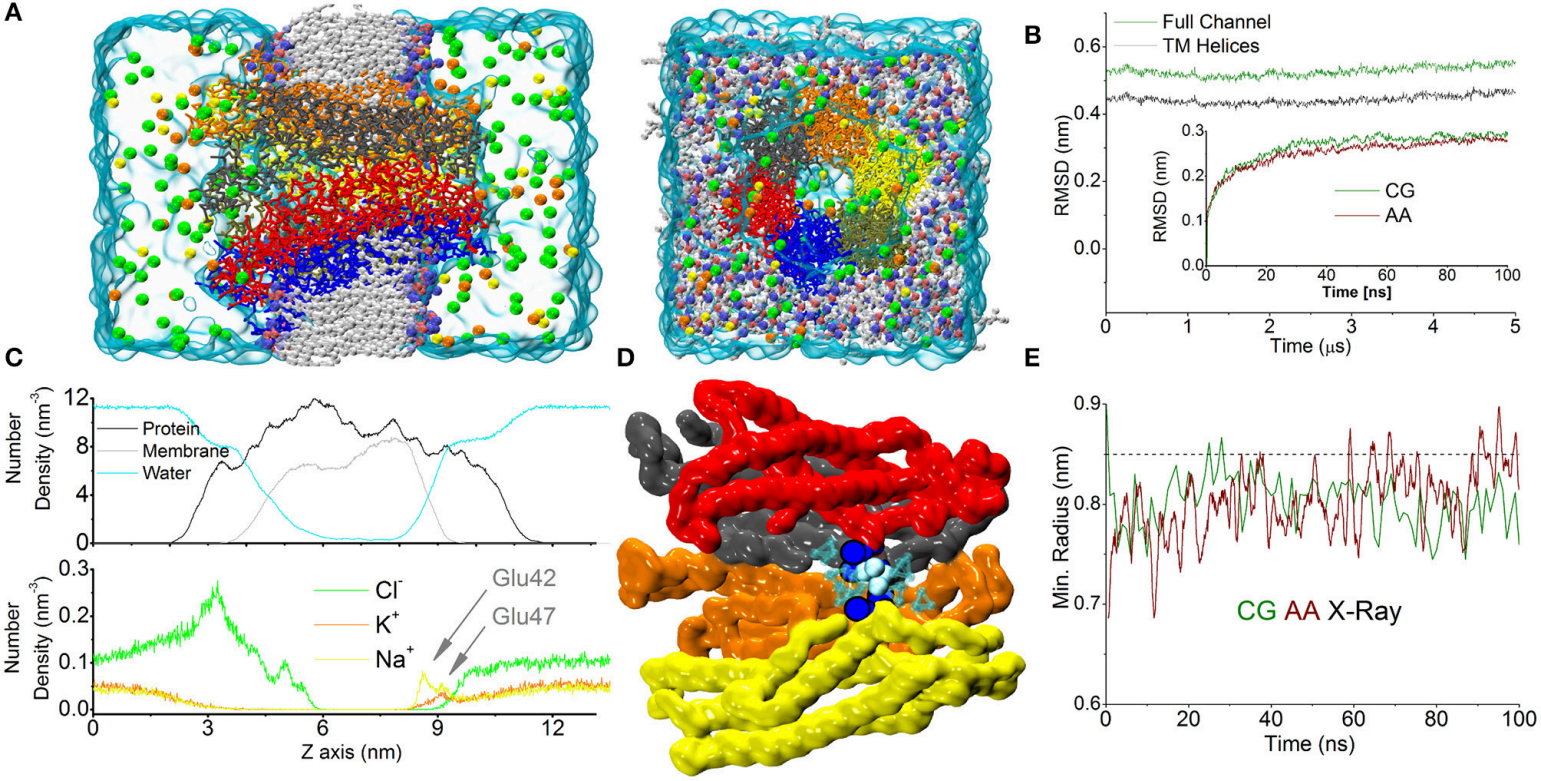
CG simulation of the Cx26 unperturbed hemichannel. **(A)** Molecular representation of the CG Connexon showing the different constituents of the computational system on side and top (extracellular) views. Electrolytes are shown with their actual solvated size. Chloride, Sodium, and Potassium are shown in green, yellow, and orange, respectively. **(B)** RMSD calculated along the dynamics on the Cα positions on the full Connexon and considering only the TM helices (dark green and black, respectively). The inset shows a comparison with the same quantity calculated from an AA trajectory during the first 100 ns of simulation reported in Zonta et al. (2015). **(C)** Lateral density profiles of different components of the system. Protein (black), membrane (including all beads, gray), and water (cyan) are shown in the top panel, while electrolytes are shown on the bottom to facilitate the visualization. Arrows indicate the cation's peaks in the density profile corresponding to the position of Glu42 and 47. **(D)** The point of maximum constriction is found at the level of Lys41 (blue spheres), consistently with the AA simulation. In this point a single coarse-grained molecule can be accommodated (cyan spheres). **(E)** Plot of minimum radius vs. time at the level of the Lys41 in the first 100 ns for CG and AA simulations (green and dark red traces, respectively). The dashed line corresponds to the X-ray structure.

The CG simulations performed as described in the Material and Methods section produced stable trajectories on a timescale spanning several microseconds. During the initial equilibration phase, the Root Mean Square Deviation (RMSD) of the whole hemichannel (shown in Figure 1A) underwent a rapid increase and stabilized near 0.55 nm (0.45 nm when considering only the TM region, Figure 1B). The increase in RMSD during the first 100 ns was mirrored by the trajectory of the AA simulation (Zonta et al., 2012) (Figure 1C, inset). This indicates that the time evolution of the CG simulation is comparable to that produced by state-of-the-art fully atomistic simulations within the time scale explored.

Calculation of the averaged density profiles of the CG system's components within the simulation box showed correct partitioning of the different species (protein, water, phospholipids, Figure 1C). In particular, Cl^−^ ions accumulated in the mouth of the channel, attracted by the numerous Lysine residues present in the intracellular loops that form the outer backbone of the cytoplasmic vestibule. In contrast, cationic species showed a marked depletion in this region. Conversely, in the extracellular vestibule, acidic residues (Glu42, 47 and Asp 46, 50) favored the accumulation of cations. In particular, the distribution of Na^+^ ions showed two peaks, roughly corresponding to the positions of Glu42 and Glu47, which coordinate six Ca^2+^ ions in the Ca^2+^-bound X-ray structure (Bennett et al., 2016). Owing to the larger size of K^+^ (compared to Na^+^), the density of K^+^ ions peaked only at the more external position of the extracellular vestibule (Figure 1C). In agreement with our published atomistic simulations, the maximal constriction of the pore corresponds to the position of the six Lys41 (Zonta et al., 2012), whose side chains leave enough room to accommodate a single CG water molecule (Figure 1D) in the center of the pore. The minimum pore diameters of the X-ray crystallographic structures, the AA and CG models are indistinguishable from each other (Figure 1E), once statistical fluctuations and experimental resolution are taken into account. In the course of this simulation, lasting 5 μs, we did not detect any net flux of water or ions across the TM region, consistent with the fact that we were simulating equilibrium conditions.

**Figure 2:**
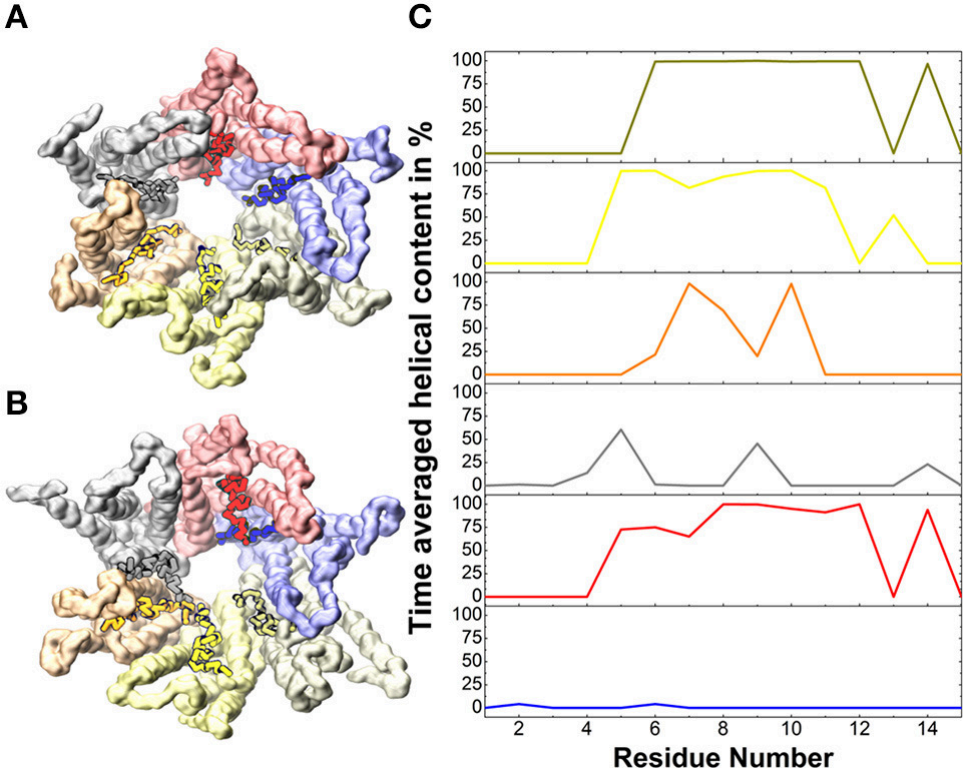
Helical content in the N-terminal helices. **(A)** Solvent accessible surface of the backbone beads of the Cx26 Connexon colored by chain for the initial conformer. **(B)** Same as **(A)** for the final conformer after 5 μs. Vivid colors indicate residues 1 to 14 on which the averaged helical content is calculated in **(C)**.

Temperature effects in atomistic simulations affect the conformation of each connexin independently, resulting in a loss of symmetry (Kwon et al., 2011). This is particularly evident at the N-terminal helices, where amino acids 1 to 14 display a high mobility with averaged helical contents varying from nearly 0% for one chain to ~80% for others (Figure 2).

Altogether, these results suggest that the conformations sampled by the CG model in a multi-μs timescale equilibrium simulation are consistent with the experimental structures, and supply a dynamical description comparable to that provided by AA models.

### External electric fields reshape the hCx26 hemichannel structure and widen its pore

**Figure 3:**
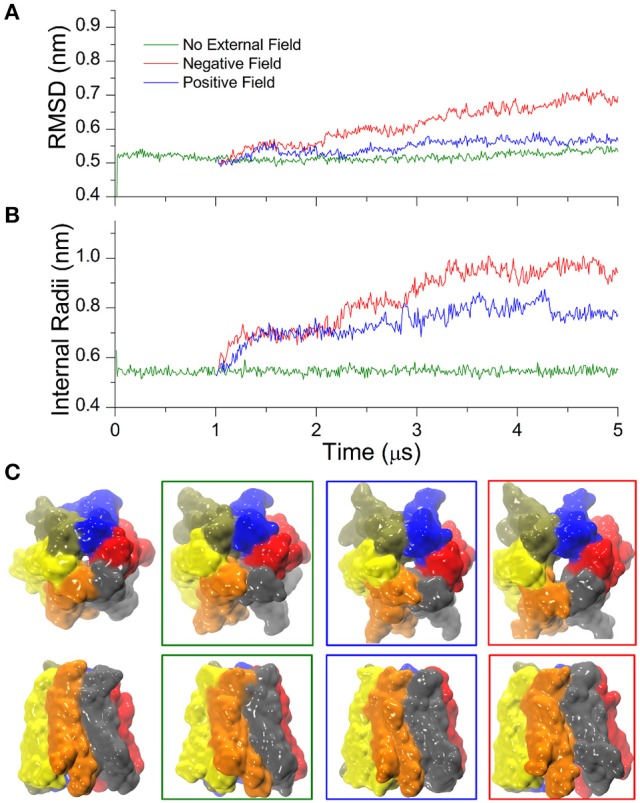
Cx26 under external electric field. **(A)** RMSD on the Cx26 Connexon in absence (green) and presence of an external field (red: negative or polarizing; blue: positive or depolarizing). The external field in either direction was applied after 1 μs of simulation in the absence of an external field. The green trace corresponds to the same simulation presented in Figure 1, which is shown here as a reference. **(B)** Time series of the maximum constriction size at the pore lumen measured at the level of Lys41 in the absence and in the presence of external field. **(C)** Solvent accessible surfaces of initial (left) and final conformers colored by chain in top and side views. The color code of the external frames is the same as in **(A,B)**.

In time-resolved X-ray crystallography, electric field pulses of about 1 MV/cm have been successfully applied to protein crystals to promote conformational changes throughout the protein structure and observe them in spatial and temporal detail (Hekstra et al., 2016). Building upon this idea, we sought to explore possible alternative conformations of the hCx26 hemichannel, by imposing *in silico* a constant electric field in the direction of the pore axis, and performed CG simulations starting from the configuration of the equilibrated system described in the previous section. In the following, we denote as “positive” an electric field that drives a cation from the cytoplasmic to the extracellular side of the plasma membrane and “negative” in the opposite direction. Furthermore, for the sake of convenience, we will hereafter express the electric field in V/nm units (1 MV/cm = 0.1 V/nm). We explored a range of electric field amplitudes around those used in Hekstra et al. (2016) and determined that values of approximately ± 0.1 V/nm resulted in a rapid (from few ns up to 1 μs) destruction of the molecular structure and membrane electroporation (Supplementary Movie SM1). Amplitudes between ± 0.1 and 0.07 V/nm resulted in conformational distortions, which included the separation of the connexin chains within few μs. These results can be in phenomenological agreement with electrophysiology experiments, since high TM potentials above ~180–200 mV invariably destroy biological membranes (Teissié and Rols, 1993). At the other extreme of the scale, electric field amplitudes below ± 0.03 V/nm did not elicit any evident effect on the structure of the hCx26connexon, within a timescale of 10 μs. In contrast, we observed a more interesting behavior for intermediate values of the electric field, i.e., induction of mild and reproducible conformational changes, which depended on the polarity of the applied field (see Figure 3 for a summary). Specifically, a negative field of 0.06 V/nm produced a smooth increase of the RMSD (Figure 3A, red trace) within few hundred nanoseconds, accompanied by an increase in the minimum internal radius of the hemichannel measured at the level of Lys41 (Figure 3B, red trace). A positive electric field of equal amplitude produced milder effects on the overall structure of the hemichannel and elicited a smaller increase in the minimum radius, mostly due to rotation of Lys41 side chains (Figure 3B, blue trace). Comparison of the solvent accessible surfaces between initial and final conformers in all three simulations (Figure 3C) shows that the small opening present in the X-ray structure at the level of the six Lys41 was maintained in the presence of the positive field, vanished in the absence of an electric field and increased upon application of a negative electric field (Figure 3C).

It is worth emphasizing that the observed relatively fast (μs) configuration transition must not be confused with the “loop gating” mechanism, as the latter closes the hemichannel for negative potentials and occurs on a far longer time scale (ms) (Sanchez et al., 2013, 2016). The transition uncovered by our CG simulations, instead, leads the hemichannel to a new stable configuration distinct from the crystallographic structures. Repeating the simulation using slightly different starting conditions reproduced qualitatively the open conformation, underlining the robustness of our approach (Figure SM1).

### Ca^2+^ inhibits the pore widening of the hemichannel

**Figure 4:**
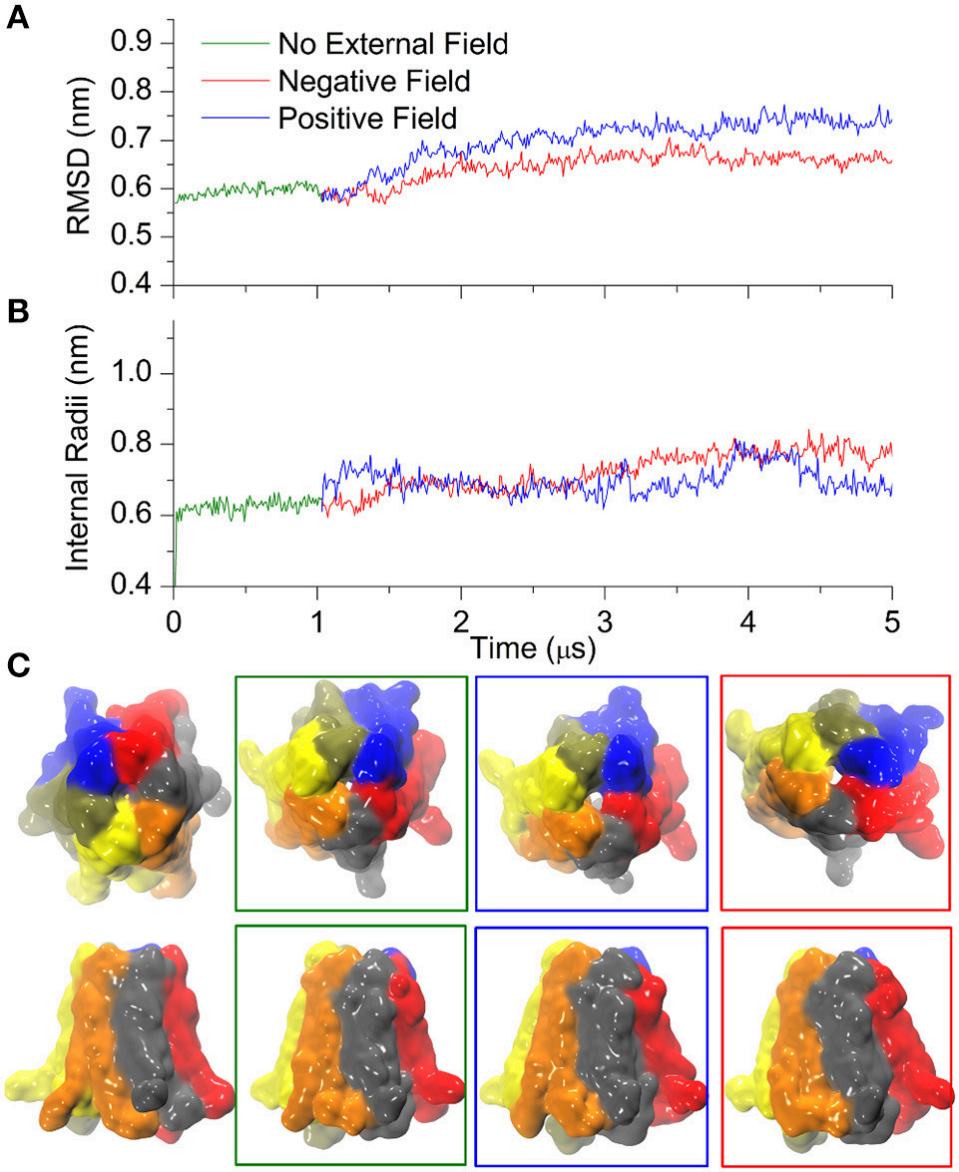
Calcium bound Cx26 under external electric field. **(A)** RMSD on the Cx26 Connexon in absence (green) and presence of an external field (red: negative or polarizing; blue: positive or depolarizing). The external field in either direction was applied after 1 μs of simulation in the absence of an external field. **(B)** Internal radii as a function of the simulation time in the absence and in the presence of external field measured at the level of Lys41. **(C)** Solvent accessible surfaces of Cx26 conformers at different point of the simulations. From left to right: initial, after 1 μs in absence of external field, after 4 μs under depolarizing field, and after 4 μs of polarizing field. The apparent differences in the external diameter are originated by the flexible C-terminal tails.

It is well-established that extracellular Ca^2+^ ions stabilize the closed state of connexin hemichannels (Verselis and Srinivas, 2008). To explore this effect we started from the Ca^2+^ bound crystal structure of the hCx26 hemichannel and used CG simulations to follow its dynamics for 1 μs in absence of external field. Thereafter we applied either positive or negative electric fields for 4 μs (± 0.06 V/nm, respectively). Trajectories were stable in the absence of electric fields, although characterized by a slightly higher value of RMSD compared with Ca^2+^-free simulations (compare Figure 4A and Figure 3A; these small differences can be ascribed to the different initial configurations of Ca^2+^-free and Ca^2+^-bound simulations).

**Figure 5:**
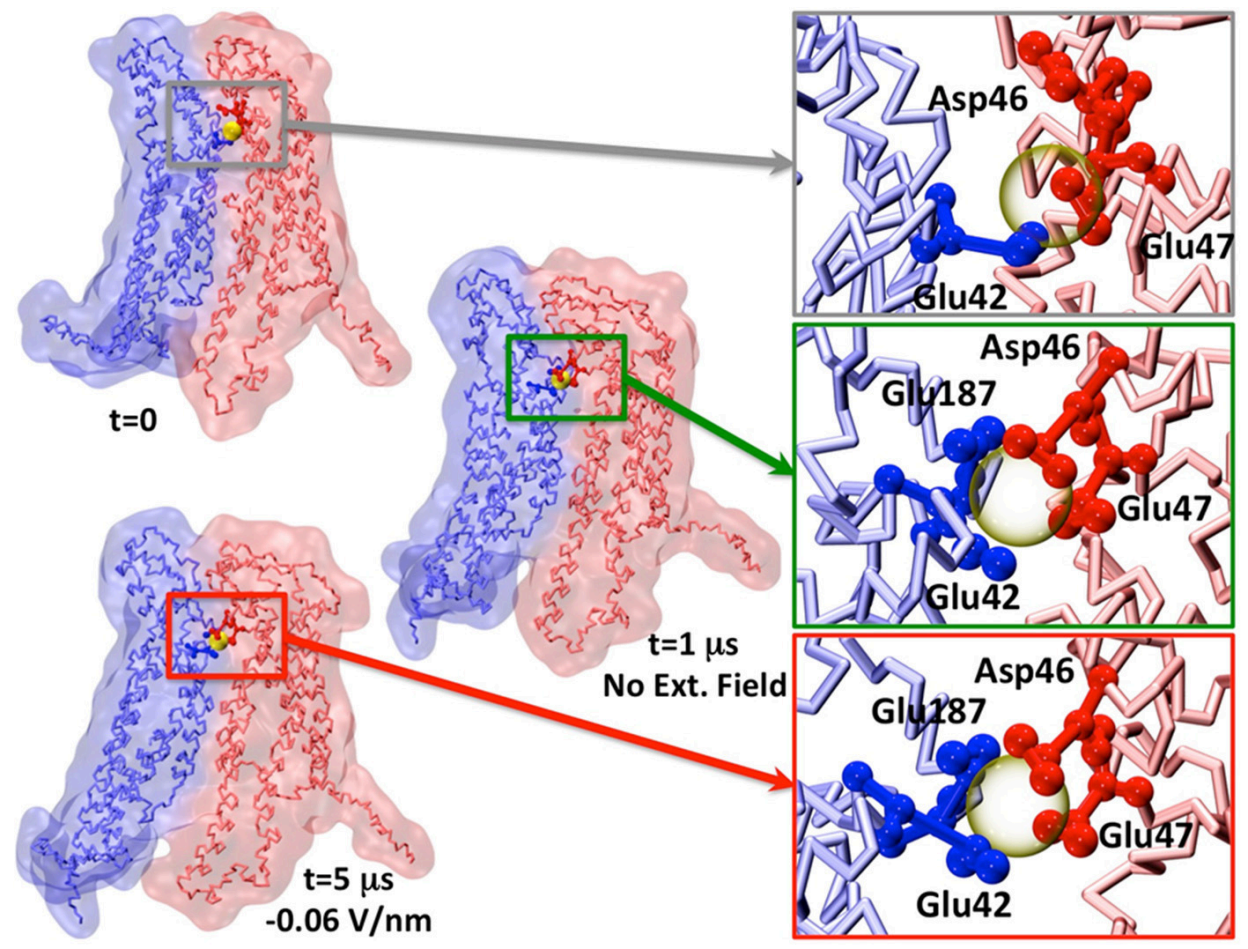
Representative snapshot of two apposed connexins coordinating one Calcium ion. The insets show the change in Calcium coordination. For the sake of visual clarity, only the amino acids within 0.5 nm of Calcium (semitransparent yellow sphere) are shown and colored by chain.

The introduction of the external field in either direction caused a small increase in RMSD that stabilized after ~3 μs (Figure 4A), whereas the internal radii remained rather insensitive to the perturbation (Figure 4B), in line with the stabilizing role of Ca^2+^. Indeed, only a small orifice is observed in both cases (Figure 4C) at the level of the Lys41 girdle, which did not allow the permeation of any coarse grained ions (which are equivalent to hydrated ions of AA simulations). Therefore, these computational results suggest that Ca^2+^ coordination within the extracellular vestibule of the channel (Figure 5) inhibits the transition to the wider pore state described in the previous paragraph.

We also notice that Ca^2+^ coordination was lost during the dynamics already during the equilibration phase (Figure 5), in agreement with previous AA simulation results (Bennett et al., 2016; Lopez et al., 2016). This is likely due to the low affinity of Ca^2+^ for the extracellular domain (in the millimolar range, Gómez-Hernández et al., 2003).

### Pore widening of the Ca^2+^-free structure depends on TM2 rearrangement at the level of Pro87

To understand the molecular mechanism leading to the widened pore configuration of the hemichannel, we examined in further detail the transition pathway. We thus noted that the final state achieved after 5 μs of CG simulation, in the presence of the negative field and in the absence of Ca^2+^, was asymmetrical and that the six protomers responded differently to the applied field during the dynamics. Moreover, RMSD computed along the transition for each protomer remained below 0.3 nm. This figure should be compared to the 0.55 and 0.75 nm RMSDs of the whole hemichannel in absence and presence of a negative electric field, respectively, indicating that the change in quaternary structure exceeded that of the individual subunits.

**Figure 6:**
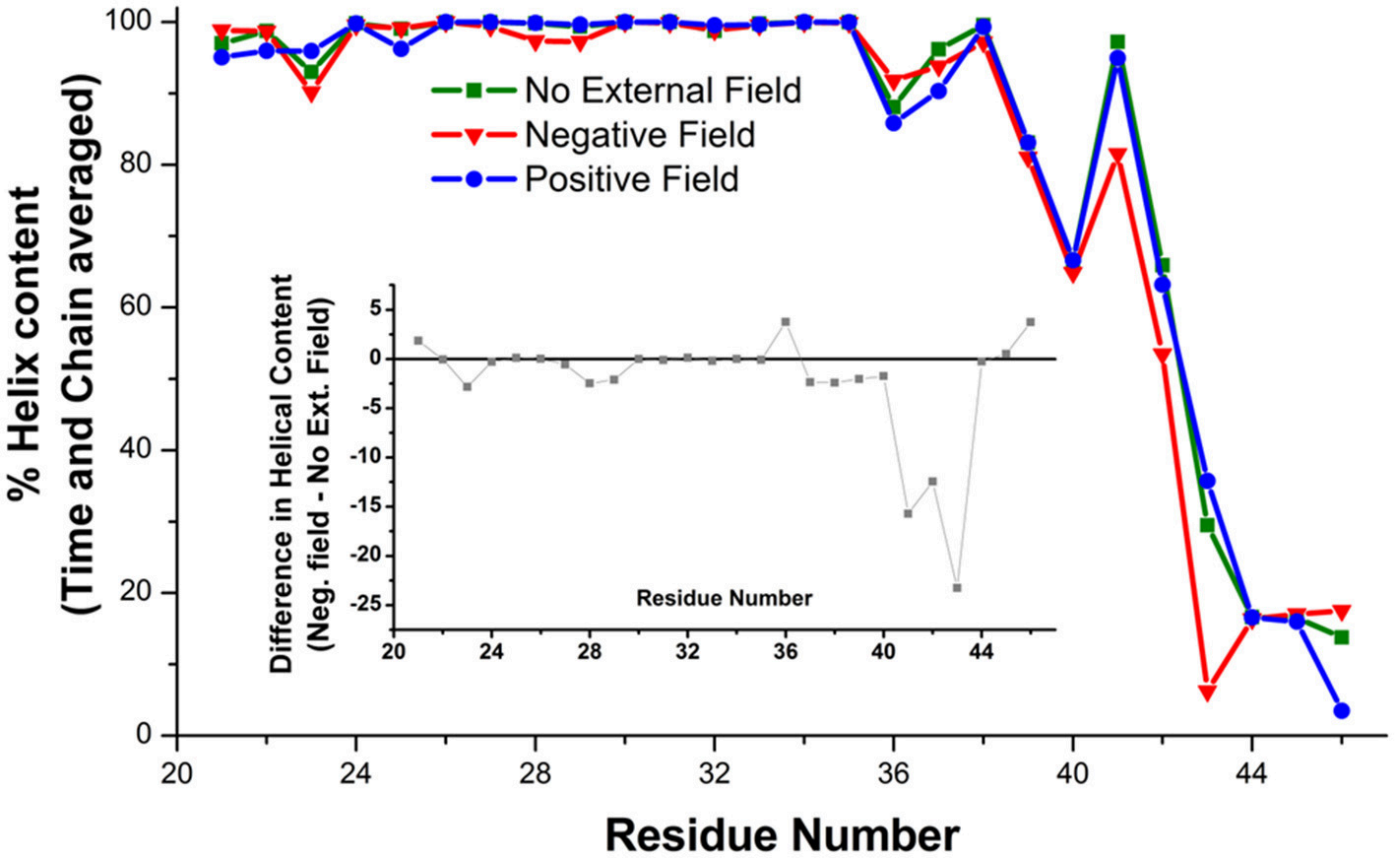
Helical content on the amino acids of the TM1 averaged on six Connexin chains and over the last μs of the simulation. The inset shows the difference between the simulations in absence (green squares) and presence (red triangles) of a hyperpolarizing electric field.

**Figure 7:**
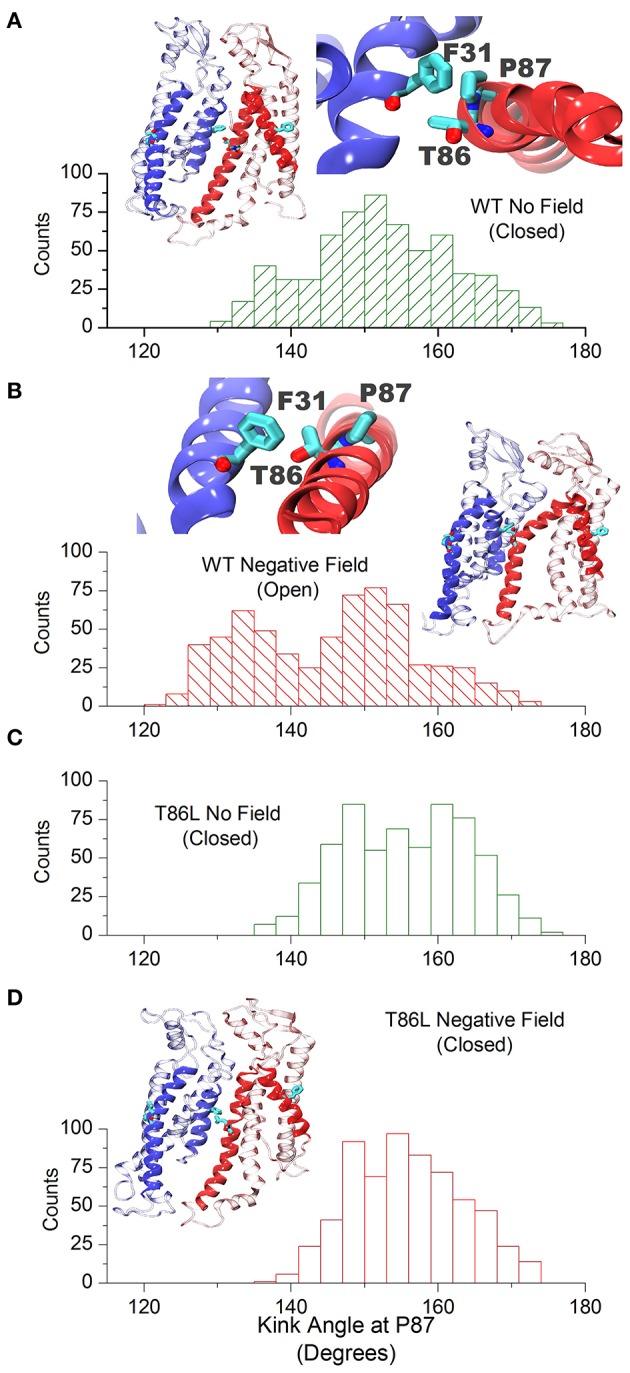
Cx26 in the open conformation. **(A)** Angle distribution of the kink at TM2 in the closed hemichannel in absence of electric field measured along the last μs of simulation. The molecular drawings show two adjacent backmapped connexins (left) and the interaction between Phe31, Thr86, and Pro87 looking from the extracellular side (right). **(B)** Same as **(A)** for the open configuration. **(C,D)** Angle distribution of the kink at TM2 for the Thr86Leu mutated Cx26 in the absence and presence of a polarizing field, respectively. The molecular drawing in **(D)** corresponds to backmapped, mutated connexins (to be compared with those in **A,B**).

At the level of the single connexin, amino acids 41 to 43 experienced a reduction in the average alpha helix content between 12 and 23% in the presence of a negative field (Figure 6). This effect does not reflect a stable conformational change but an increased structural disorder, in agreement with the proposal that this region behaves like a labile “parahelix” (Tang et al., 2009). More importantly, we noted a shift toward lower angle values (comprised between 120 and 140°), in the TM2 kink, which is due to the presence of a conserved Pro at position 87 (Figures 7A,B). To explore this phenomenon in further detail, we used the backmapping capabilities of the SIRAH force field (Machado and Pantano, 2016) to generate pseudo-atomistic models of the initial and final CG conformers. In the backmapped conformations, the side chain of Thr86 in TM2 interacted with Phe31 in an adjacent connexin, (see insets in Figures 7A,B). Mutation of this Threonine has been shown to shift the dependence of Cx32 gap junction channels on the transcellular voltage (*V*_j_ gating) (Ri et al., 1999). Therefore, we predicted that the Thr86Leu (T86L) mutation should impair the electric field-driven conformational transition. We confirmed this prediction by performing a CG simulation on the hCx26T86L mutant. The mutated hemichannel evolved toward a more linear configuration of TM2 regardless of the application of an external field (Figures 7C,D) and remained in a state very similar to the conformation of the WT hCx26 connexon in the absence of an external field.

**Figure 8:**
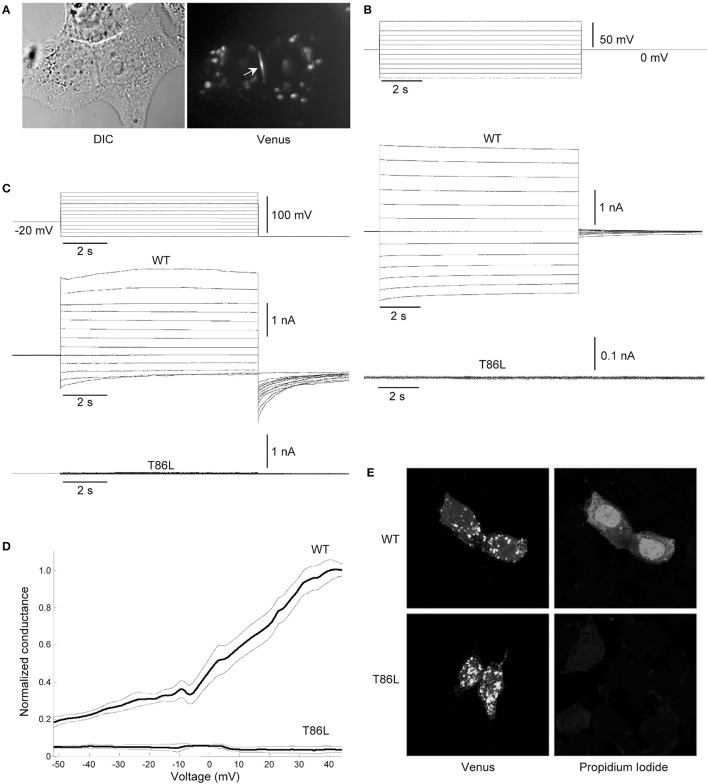
Patch clamp and dye-uptake assays in HeLa DH transfectants**. (A)** Left: differential contrast image (DIC) of HeLa DH cells transiently transfected with hCx26T86L fused in frame with the GFP color mutant Venus; right: same field of view under fluorescence illumination, highlighting a gap junction plaque between a pair of adjacent cells (arrow). Cytosolic fluorescent puncta are consistent with previous analysis of connexin trafficking en route to the plasma membrane (Thomas et al., 2005). **(B)** Voltage commands applied to cell 1 (top traces) elicited junctional currents in the adjacent cell (cell 2) of a pair of HeLa DH cells transiently trasfected with wild type (WT) hCx26 (middle traces); junctional currents were undetectable in T86L transfectants (bottom traces). **(C)** Representative measurements of hemichannel currents elicited by voltage commands (top traces) in HeLa DH cells transiently trasfected with wild type (WT) hCx26 (middle traces); hemichannel currents were undetectable in T86L transfectants (bottom traces). **(D)** Membrane conductance in response to slow (400 s) voltage ramps from nominal +60 to −60 mV; ordinates were normalized by the mean conductance of WT hCx26 at +40 mV; abscissas were corrected for the voltage drop due to the access resistance (18 ± 3 MOhm for T86L, *n* = 6; 19 ± 5 MOhm for WT, *n* = 6); measurements in **(B,C)** were performed in 0.2 mM extracellular Ca^2+^. **(E)** Incubating HeLa DH cells for 60 min in nominally Ca^2+^-free extracellular solution containing Propidium Iodide 0.25 mM promoted dye uptake in cells expressing WT hCx26 (top), whereas cells expressing the T86L mutant failed to uptake the dye (bottom).

To substantiate these computational findings, we performed patch clamp and dye-uptake assays in HeLa DH cells transiently transfected with WT hCx26 or its T86L mutant (Figure 8). Although mutant proteins trafficked correctly to the cell plasma membrane, where they also formed gap junction plaques (Figure 8A), cells remained electrically uncoupled (Figure 8B). Likewise, prominent hemichannel currents were elicited in cells expressing only the WT connexin (Figure 8C). These voltage-clamp experiments were performed in 0.2 mM Ca^2+^, a condition, which favors hemichannel opening (González et al., 2006; Sanchez et al., 2010, 2014; Fasciani et al., 2013). The absence of measurable hemichannel conductance in cells expressing the mutant connexin was confirmed by measuring the whole cell current as a function of TM potential (Figure 8D). Consistent with these findings, cells expressing the T86L mutant failed to uptake Propidium Iodide in nominally Ca^2+^-free extracellular medium, whereas cells expressing WT hemichannels readily up took the dye (Figure 8E).

## Discussion and conclusions

Achieving a reliable theoretical framework for the study and prediction of permeation properties in connexin hemichannels represents a milestone in the understanding of cell communication and membrane biophysics. While molecular dynamics simulations can be regarded, in principle, as a suitable tool, in practice they are limited by the difficulty of sampling adequately the configuration space accessible to the system composed by the hemichannel and the molecules that permeate it. In particular, AA simulations failed to reproduce any of the configuration transitions, which are expected on the basis of known gating mechanisms.

In this article, we mimicked the experimental technique proposed in Hekstra et al. (2016), and applied external electric fields on a CG representation of the hCx26 hemichannel in a phospholipid membrane. Converting the electric field module to transmembrane voltages and considering the box dimensions, the applied potential was nearly 800 mV, i.e., roughly four-fold larger than the rupture voltage for biological membranes (Chen et al., 2006). Although similarly large voltages are routinely used in simulations studies (Delemotte et al., 2011; Escalona et al., 2016), a direct comparison between the energetics of real systems with their CG representations is impossible due to the loss of degrees of freedom inherent in the coarse-graining process. Consequently, the results obtained in the present study should be considered at the qualitative level, at least as far as membrane potential is concerned.

Based on the SIRAH force field, the electric field promoted a conformational change only in the wt channel under negative field direction. Positive electric field, Calcium or an *ad-hoc* point mutation showed no sensitivity to the external field.

**Figure 9:**
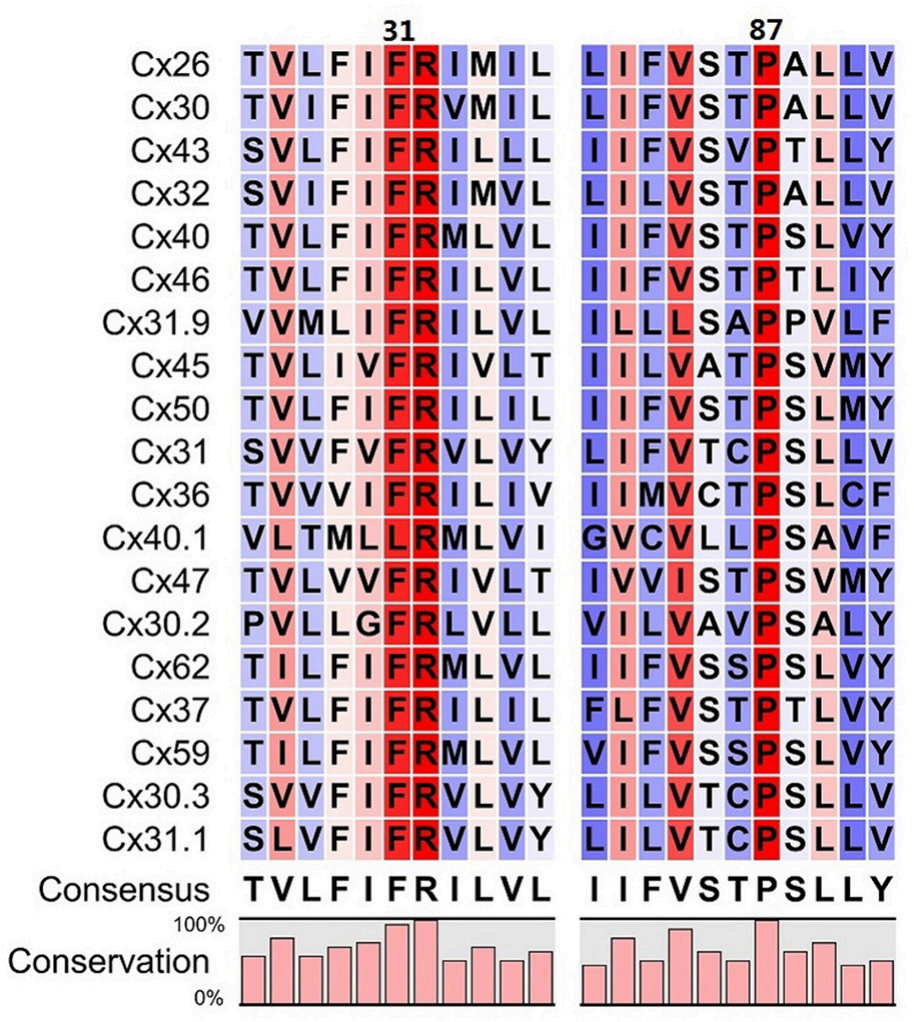
Sequence alignment around residues 31 and 87 of the 19 human connexins that are known to be translated into proteins. Colors refer to the percentage of consensus, from blue (30%) to red (100%), numbering refers to the amino acid sequence of Cx26. Amino acids in positions 86 always have a small side chain (T, A, C, V, S), with the only exception of Cx40.1 (L). In this connexin, the phenylalanine in position 31 is replaced by a leucine, whose side chain is smaller, assuring that the overall steric hindrance of the two opposed amino acids is conserved along the family.

The conformational transition toward a new stable state of the hemichannel characterized by an increased pore diameter took place within the microsecond timescale. This transition required the increase of the TM2 kink angle at the level of Pro87, which faces Phe31 in TM1. Although qualitative, our simulation scheme resulted accurate enough to predict that an amino acid bulkier than Threonine in position 86 should impair pore widening by creating steric hindrance with Phe31 (Figure 7). We verified this prediction by dye uptake experiments in HeLa cells transfected with hCx26WT and hCx26T86L mutant hemichannels (Figure 8) (Ri et al., 1999). Pro87 and Phe31 are conserved in all human connexins, with the notable exception of Cx40.1 (Figure 9). On the other hand, Thr86 is replaced only by small amino acids (i.e., Ala, Cys, Val, and Ser), suggesting that the size of the side chains in this position is a key determinant of channel function. In fact, the only connexin featuring a bulkier amino acid is, again, Cx40.1, which has a Leu instead of Thr86, suggesting that the presence of a larger side chain at position 86 must be compensated by a smaller (Phe to Leu) side chain at position 31 in order to decrease steric hindrance and to allow the conformational transition. Hence, our results suggest that the collective degree of freedom that enables the transition described above is shared among the whole connexin family.

Nevertheless, a direct connection between such electric field-driven transition and hCx26 hemichannel gating mechanisms should not be made at this point. Indeed, multiple experiments show that, in the absence of Ca^2+^ and at negative potentials, hCx26 hemichannel exhibit active loop gating events i.e., open-close transitions, which can last even seconds (see for example single channels recording in Sanchez et al., 2016 or Sanchez et al., 2013). At most, it could be speculated that the combination of the external electric field with the CG force field excited a collective degree of freedom, which might also be used by one of the hemichannel gating mechanisms.

While hinted by several experimental studies, our model shows for the first time how a connexin hemichannel can rearrange the quaternary structure in response to an external field. The cost-effective characteristic of our simulation scheme could allow for a large-scale mutagenesis studied on one or more members of the connexin family, to facilitate the rationalization of spontaneously arising mutations or polymorphisms.

## Author contributions

FZ and SP designed research. FZ, SP, and DB performed computer simulations. FB, AC, and GC performed experiments. FM designed and supervised experiments. FZ, DB, SP, GY, and FM analyzed data. FZ, SP, and FM wrote the paper.

### Conflict of interest statement

The authors declare that the research was conducted in the absence of any commercial or financial relationships that could be construed as a potential conflict of interest. The reviewer UZ and handling Editor declared their shared affiliation.

## Supplementary material

Figure SM1 Independent replicas of the Cx26 under external electric field**. (Top)** RMSD on the Cx26 hemichannel in absence (green) and presence of an external field (red: negative; blue: positive). The two columns identify independent replicas. The external field in either direction was applied immediately after the stabilization period re-generating a temperature distribution using different random seeds. **(Bottom)** time dependence of the internal radii measured at the level of Lys41.

Supplementary Movie SM1 Excessively high electric fields destroy protein and membrane systems. The simulation was carried out applying an external field in the -z direction during 100 ns.
