# Supplementary figures and images for: Cues to Opening Mechanisms From in Silico Electric Field Excitation of Cx26 Hemichannel and in Vitro Mutagenesis Studies in HeLa Transfectans
Source: Front Mol Neurosci. 2018 May 31;11:170. doi: 10.3389/fnmol.2018.00170 (PMC5990870; doi:10.3389/fnmol.2018.00170)

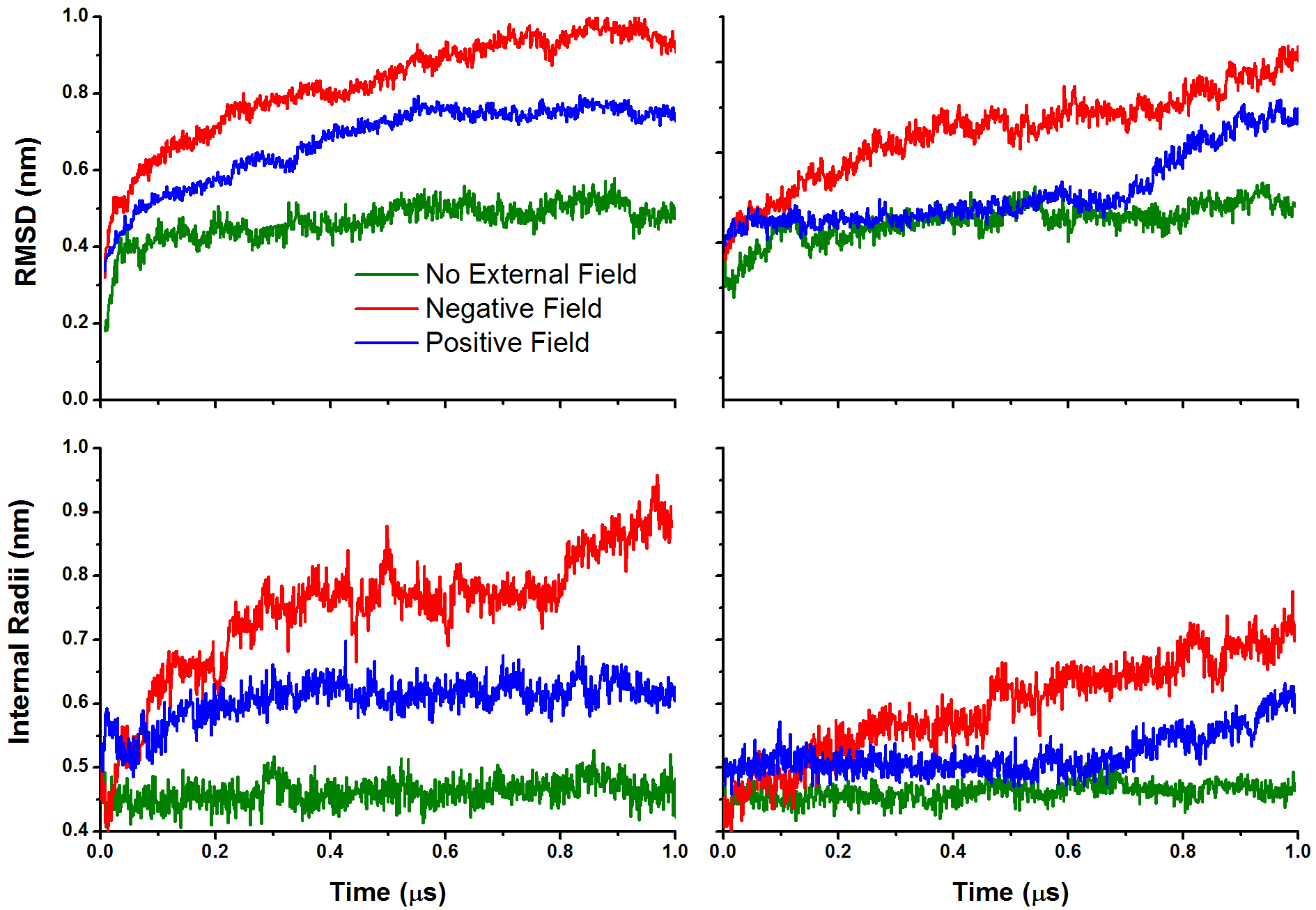

Supplement: Figure SM1 — Independent replicas of the Cx26 under external electric field. (Top) RMSD on the Cx26 hemichannel in absence (green) and presence of an external field (red: negative; blue: positive). The two columns identify independent replicas. The external field in either direction was applied immediately after the stabilization period re-generating a temperature distribution using different random seeds. (Bottom) time dependence of the internal radii measured at the level of Lys41. [file Image_1.PNG]
